# Finding of a mass on the mitral valve in a patient on chronic dialysis

**DOI:** 10.1016/j.radcr.2025.01.020

**Published:** 2025-02-01

**Authors:** Vasil Papestiev, Marjan Shokarovski, Nikola Lazovski, Nadica Mehmedovic, Valentina Andova, Gordana Petrushevska, Ljubica Georgievska-Ismail

**Affiliations:** a University Clinic for Cardiac Surgery, Medical Faculty, Ss. Cyril and Methodius University of Skopje, Majka Tereza no. 17/building 8, Skopje 1000, Republic of North Macedonia; b University Clinic of Cardiology, Medical Faculty, Ss. Cyril and Methodius University of Skopje, Republic of North Macedonia, Majka Tereza no. 17/building 8, Skopje 1000, Republic of North Macedonia; c Institute of Pathology, Medical Faculty, Ss. Cyril & Methodius University of Skopje, Republic of North Macedonia, 50 Divizija, 6b, Skopje 1000, Republic of North Macedonia

**Keywords:** Mitral valve mass, Echocardiography, Myxoma, Endocarditis

## Abstract

Myxomas are cardiac neoplasms that are most commonly located in the left atrium, usually arising from the vicinity of the fossa ovalis. However, there have been cases, although very rarely, of valvular myxoma. A cardiac mass found incidentally on echocardiography can present a challenge in particular if asymptomatic or found in an unusual location. We present the case of a 58-year-old male with kidney disease treated with chronic dialysis, referred to the cardiology clinic because of an incidental finding of a mitral valvular mass on routine transthoracic echocardiography. Although this lesion was initially misdiagnosed as native valvular endocarditis with vegetation, a series of clinical and radiological investigations led to the preoperative diagnosis of possible papillary fibroelastoma or calcified thrombotic mass. Given the increased risk of embolization due to the mass being mobile and greater than 1 cm in size, the patient was referred to cardiac surgery. Excision of the mass without mitral valve replacement was performed. Histopathological findings of the mass revealed the existence of a cardiac myxoma. In such cases of a mitral valve mass, multimodality imaging should have of high priority to achieve an accurate diagnosis. Although a definitive diagnosis can only be established after surgical excision of the mass and histopathological confirmation, it is very important to consider a differential diagnosis of mitral valve myxoma in any patient with an unexplained mitral valve mass.

## Introduction

Intracardiac masses located at the valves are predominately suspected to be papillary fibroelastoma, vegetation, a thrombus, a Lambl's excrescence, and rarely a cardiac myxoma[[1], [2], [3]]. These all possess characteristic findings which along with the clinical circumstances can help differentiate them from each other and help avoid a misdiagnosis.

In particular, diagnosiscan be challenging if such a cardiac mass is asymptomatic, found in an unusual location and is incidentally found on echocardiography [[4], [5], [6], [7]]. To avoid misdiagnosis, imaging methods such as a computed tomography scan (CT) and/or cardiac magnetic resonance imaging (MRI) should be used [8]. Given that these lesions are usually benign, but can often cause serious complications, surgical management is required [[9], [10], [11], [12]].

## Case description

A 58-year-old male was referred to the cardiology clinic because of an incidental finding of a mitral valvular mass on a routine annual transthoracic echocardiography (TTE) at a private physician's office. On admission, the patient was asymptomatic, with regular heart rate and rhythm and without murmurs on physical examination. Relevant past medical history included hypertension andchronic kidney disease (CKD) treated with chronic dialysis. Given the patient's history of CKD and chronic dialysis, the mobile mitral valve mass (12 × 17 mm) observed on 2D TTE on admission, was interpreted as highly suspicious vegetation on the atrial side of the anterior mitral leaflet, in spite of it's movement which was in the same direction as the valve.

The patient was then admitted to the valvular disease department with the diagnosis of endocarditis and empiric antibiotic therapy was started immediately.

During the 2 weeks' stay in the hospital, the patient's ECG was continuously normal and his laboratory studies were unremarkable for any inflammatory markers. Repeat blood culture remained free of microbial growth and the patient exhibited no symptoms consistent with overt endocarditis. The consecutive echocardiographic assessments did not show any improvement in the size of the mitral mass or the severity of mitral regurgitation. However, during the hospital stay, no further examinations were done to confirm the referral diagnosis.

**Fig. 1:**
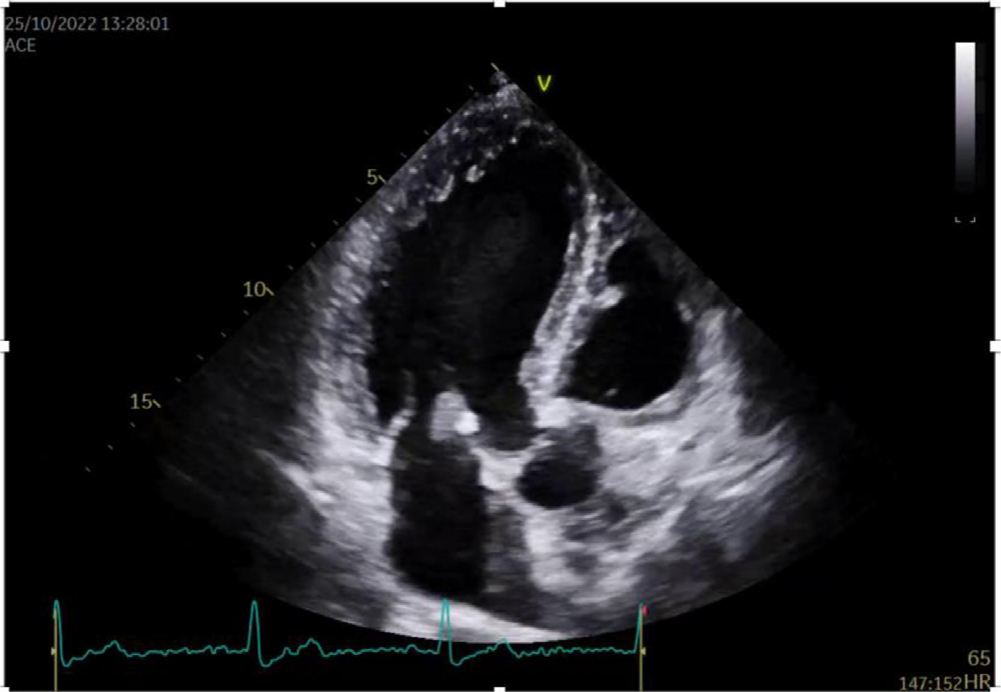
2D Transthoracic echocardiography image in the PLAX view showingg a globular mass attached to the mitral valve.

**Fig. 2:**
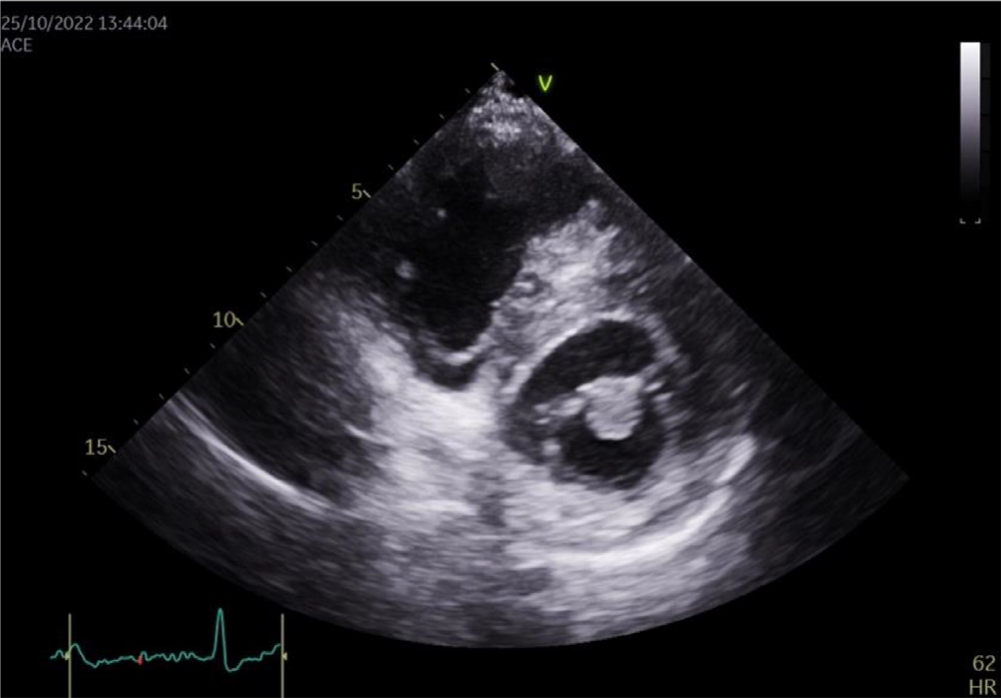
2D Transthoracic echocardiography image in the PSAX view showing a globular mass attached to the mitral valve.

**Fig. 3:**
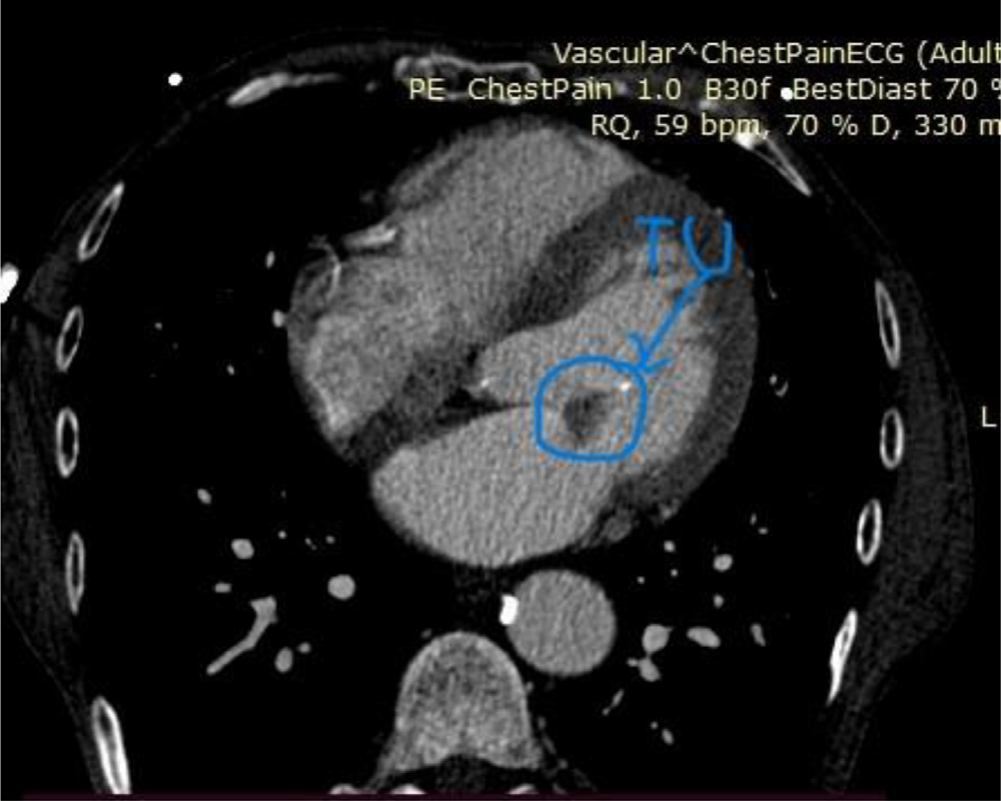
CT imaging of a tumorous mass at the mitral valve.

At this time, the patient was asymptomatic and feeling well, but with respectable fear about the uncertainty of his diagnosis and prognosis, and proceeded to request a second cardiology opinion. A follow-up 2DTTE was performed and the mass was confirmed to be 1.5 cm in size, mobile, attached to the anterior mitral leaflet, and round with a homogeneously speckled surface without significant mitral regurgitation or obstruction. (Fig. 1, Fig. 2) Differential diagnoses at this time included papillary fibroelastoma or calcified thrombotic mass. To confirm the diagnosis, a CT was ordered, but the result was inconclusive. (Fig. 3) Given the increased risk of embolization with mitral valve mobile masses greater than 1cm in size, we referred the patient to cardiac surgery which was done immediately.

**Fig. 4:**
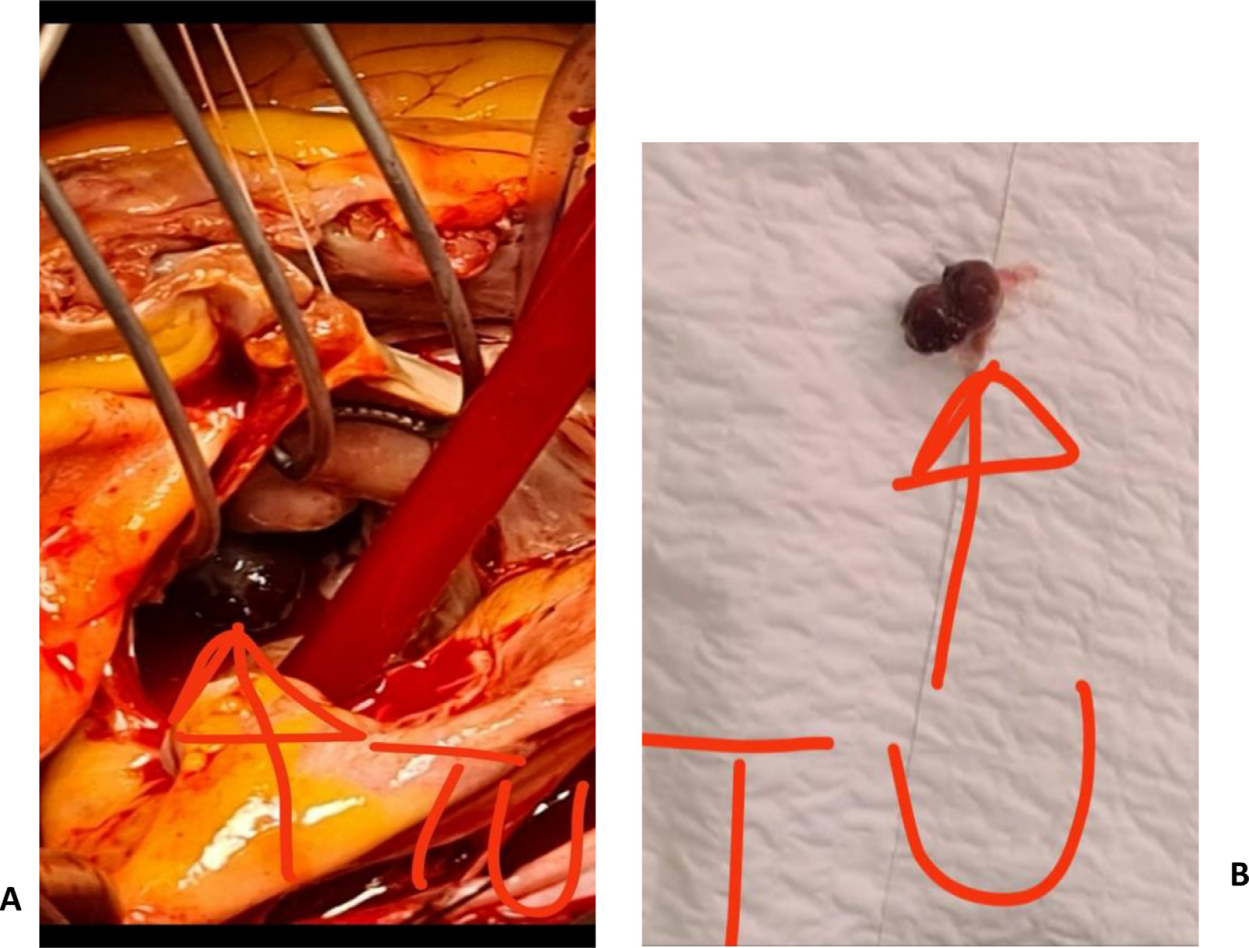
(A) Operative view of the rounded mass attached to the mitral valve and (B) Macroscopic view of the operative spacemen.

During the operation, a median sternotomy was performed. After full heparinisation and standard aortic and bicaval cannulation, cardiopulmonary bypass was started. The heart was arrested via antegrade cold blood cardioplegia. Left atriotomy was performed in the Sondergaard's groove and the mass was easily identified on the anterior mitral valve leaflet adjacent to the mitral valve annulus. The mass was excised in the sub-endocardial layer without damaging the anterior mitral valve leaflet (Fig. 4). Тhere was no need for additional anterior mitral valve leaflet reconstruction or mitral annuloplasty. After closing the left atrium and appropriate de-airing, the patient was weaned from cardiopulmonary bypass. ECG confirmed normal sinus rhythm. Anticoagulation was reversed with protamine and his chest was closed after placement of drains and pacing wires. After the operation, TTE confirmed appropriate mitral valve function with no evidence of a mass or mitral valve regurgitation.

**Fig. 5:**
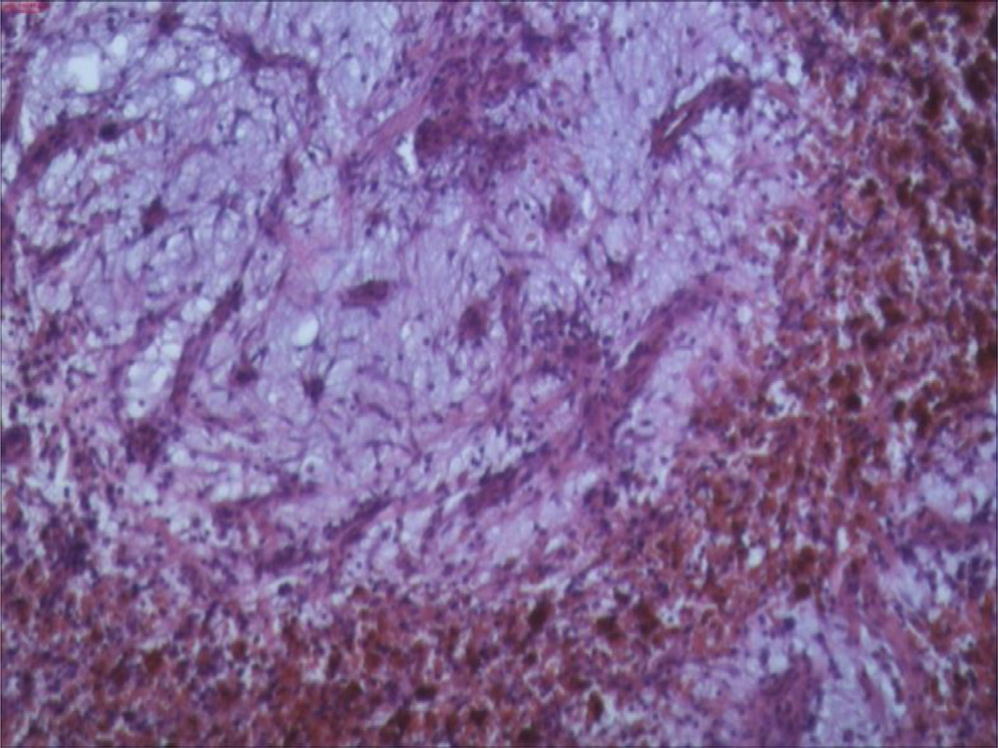
Myxoma cells isolated or arranged in cords and scattered within the myxoid stroma. A hemorrhage is seen with hemosiderin deposits adjacent to the tumor tissue (Gandy – Gamna bodies) (H&E, 200x; NIKON Eclipse 80).

**Fig. 6:**
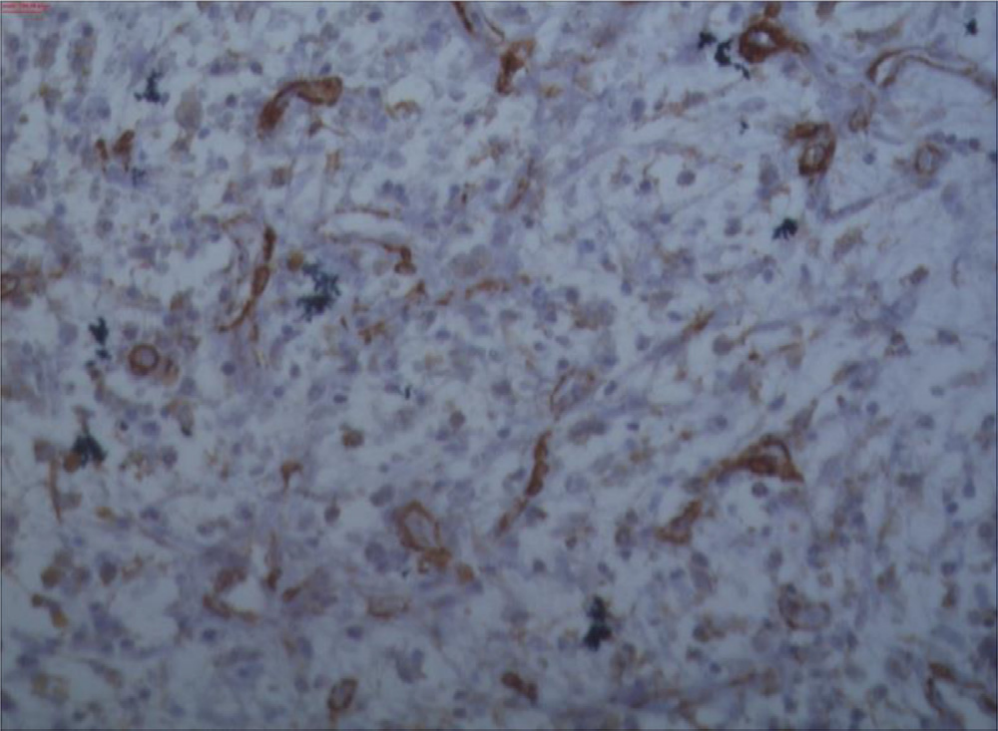
S100 immunoreactivity in myxoma cells scattered within the myxoid stroma (PT LINK Immunoperoxidase) (200x, NIKON Eclipse 80).

Postoperative histopathological findings revealed tissue sections made up of myxoid stroma with embeded stellate cells and cells with fibroblastoid morphology, findings that favor the existance of cardiac myxoma (Fig. 5, Fig. 6).

The patient's postoperative course was uneventful. The patient was discharged home with regular yearly follow-up with the cardiologist.

## Discussion

Myxoma is a benign tumor that usually arises in the left atrium; however, there have been rare cases of its occurrence in the left ventricle or right atrium and very rarely on the mitral valve [[4], [5], [6], [7]]. The clinical presentation of a myxoma can vary widely and generally depends on the size and location of the tumor within the heart. It may present with symptoms ’such as intracardiac obstruction, constitutional symptoms, and/or symptoms of ischemia or infarction due to the embolization, but it can also be asymptomatic and be an incidental echocardiographic finding representing a real challenge for diagnosis and treatment.

Thus, the dilemma in everyday practice is how to differentiate between cardiac masses located at the valves, mainly at the mitral valve, especially in specific clinical circumstances that could favor 1 diagnosis over another.

Differentiating infective endocarditis (IE) with vegetations from myxomas has been proven to be quite difficult, especially in patients predisposed to IE as was the index case of the man with CKD treated with chronic dialysis [[13], [14], [15], [16]]. It should be emphasized that in order to avoid misdiagnosis in everyday practice, in the absence of fever, the presence of a negative blood culture, and lack of systemic embolism, such large vegetation should always be investigated as a potential tumor mass.

However, the echocardiographic features of the valvular mass should also be rather helpful in differentiating vegetation from myxoma. Unlike vegetation, myxomas have a more regular shape, are more defined and more echogenic compared to the surrounding structures, and have more homogeneous density. Additionally, myxomas move together with the cusps, prolapse in the diastole in the left ventricle, and return to the left atrium in systole, and importantly, there is no destruction of the valve thus mitral regurgitation is absent [[2], [3], [4], [5], [6], [7], [8]].

Given this was a patient with CKD, a calcified amorphous tumor (CAT) or calcified thrombotic mass were strongly considered [6,17,18]. Considering that the CT results did not add any clarification to the puzzle, papillary fibroelastoma was the suspected diagnosis by the clinical and surgical teams [6,19].

As for the decision to operate on an asymptomatic patient with a mass at the mitral valve who is on chronic dialysis, there are no clear guidelines on the management in these cases. It is well known that patients on chronic dialysis have increased odds of postoperative mortality [20], but given that the mass was left-sided, mobile, and more than 1cm in size and highly prone to embolization and complications, surgical management was required [2,9]. Fortunately, the patient's surgical intervention and postoperative course was without any complications, which justified our decision.

We have to confess, that the histopathological finding of cardiac myxoma was a surprise for the cardiology team, but we considered it as a lesson for future decisions: mainly to take all existing clinical information and multimodality imaging [8] into account, and not be overly influenced by the primary disease which could lead to misdiagnosis.

## Conclusion

The finding of a cardiac mass at the mitral valve has multiple implications, in particular in patients with chronic kidney disease who are on hemodialysis. While most often this finding can be infective endocarditis with vegetations, differential diagnoses include a calcified amorphous tumor or calcified thrombotic mass, papillary fibroelastoma, and very rarely a cardiac myxoma. Clinical circumstances especially multimodality imaging should be prioritized in order to reach the proper diagnosis. However, the final diagnosis can only be established after surgical excision of the mass and histopathological confirmation.

## Patient consent

Written informed consent for the publication of this case report was obtained from the patient.
